# PRMT6 diminishes HIV-1 Rev binding to and export of viral RNA

**DOI:** 10.1186/1742-4690-3-93

**Published:** 2006-12-18

**Authors:** Cédric F Invernizzi, Baode Xie, Stéphane Richard, Mark A Wainberg

**Affiliations:** 1 McGill University AIDS Centre, Lady Davis Institute for Medical Research, Sir Mortimer B. Davis Jewish General Hospital, 3755 Côte-Ste-Catherine Rd, Montréal, Québec H3T 1E2, Canada; 2 Terry Fox Molecular Oncology Group and Bloomfield Centre for Research on Aging, Lady Davis Institute for Medical Research, Sir Mortimer B. Davis Jewish General Hospital, 3755 Côte-Ste-Catherine Rd, Montréal, Québec H3T 1E2, Canada

## Abstract

**Background:**

The HIV-1 Rev protein mediates nuclear export of unspliced and partially spliced viral RNA through interaction with the Rev response element (RRE) by means of an arginine rich motif that is similar to the one found in Tat. Since Tat is known to be asymmetrically arginine dimethylated by protein arginine methyltransferase 6 (PRMT6) in its arginine rich motif, we investigated whether the Rev protein could act as a substrate for this enzyme.

**Results:**

Here, we report the methylation of Rev due to a single arginine dimethylation in the N-terminal portion of its arginine rich motif and the association of Rev with PRMT6 *in vivo*. Further analysis demonstrated that the presence of increasing amounts of wild-type PRMT6, as well as a methylation-inactive mutant PRMT6, dramatically down-regulated Rev protein levels in concentration-dependent fashion, which was not dependent on the methyltransferase activity of PRMT6. Quantification of Rev mRNA revealed that attenuation of Rev protein levels was due to a posttranslational event, carried out by a not yet defined activity of PRMT6. However, no relevant protein attenuation was observed in subsequent chloramphenicol acetyltransferase (CAT) expression experiments that screened for RNA export and interaction with the RRE. Binding of the Rev arginine rich motif to the RRE was reduced in the presence of wild-type PRMT6, whereas mutant PRMT6 did not exert this negative effect. In addition, diminished interactions between viral RNA and mutant Rev proteins were observed, due to the introduction of single arginine to lysine substitutions in the Rev arginine rich motif. More importantly, wild-type PRMT6, but not mutant methyltransferase, significantly decreased Rev-mediated viral RNA export from the nucleus to the cytoplasm in a dose-dependent manner.

**Conclusion:**

These findings indicate that PRMT6 severely impairs the function of HIV-1 Rev.

## Background

Human immunodeficiency virus type 1 (HIV-1) encodes a 116 amino acid regulator of viral protein expression termed Rev. This protein is found in the nucleolus, the perinuclear zone and the cytoplasm of infected cells [1,2]. A two-exon version of Rev is translated from fully spliced viral RNA during early stages of viral replication and mediates nuclear export of unspliced and partially spliced HIV-1 RNA [2]. Rev interacts with the *cis*-acting Rev response element (RRE) located in the *env *gene [3]. Shuttling of Rev between nucleus and cytoplasm is dependent on several cellular proteins, e.g. eIF-5A, nucleoporins (Rip/Rab), CRM1, Ran-GTP, importin-β and Sam68 [1,4-11]. Different sequence motifs of Rev are important for its activity: the leucine rich motif (LRM) located in the C-terminal domain contains a nuclear export signal (NES), whereas the arginine rich motif (ARM) within the N-terminal portion of Rev harbors a nuclear localization signal (NLS) and is responsible for binding to the RRE as well as for Rev nucleolar localization [1,4]. Phosphorylations (positions S5, S8, S54/S56, S92, S99, S106) are the only type of posttranslational modifications that have been reported for Rev and are not required for its biological activity; however, these events might play a regulatory role in helping to govern viral replication [3,12-14].

There is strong evidence that Rev contains a helix-loop-helix secondary structure and that the ARM is part of the second helix [15]. The ARM contains four major amino acids (R35, R39, N40 and R44) that participate in base-specific contacts with the high affinity binding site of the RRE [1,16]. In addition, the ARM is flanked by multimerization sites at which interaction between multiple Rev proteins is thought to take place during the binding of a single molecule of viral RNA [1]. Multimers of Rev have been described in the nucleolus as well as the cytoplasm [17] and there are reports about structural transitions of Rev that appear to exist in monomeric form as a molten globule versus a more compact structure when Rev is multimerized [18]. One group has demonstrated that Rev multimerization can be dispensed with if Rev contains additional basic residues [19]. It has also been reported that Rev function is non-linear with respect to the intracellular concentration of Rev needed for multimerization [1] and that the sensitivity of HIV-1 infected primary T cells to killing by cytotoxic T lymphocytes (CTL) is determined by Rev activity [20]. As a consequence, it has been proposed that low levels of Rev can lead to a state of proviral latency in CD4+ memory T cells [21,22].

**Figure 1:**
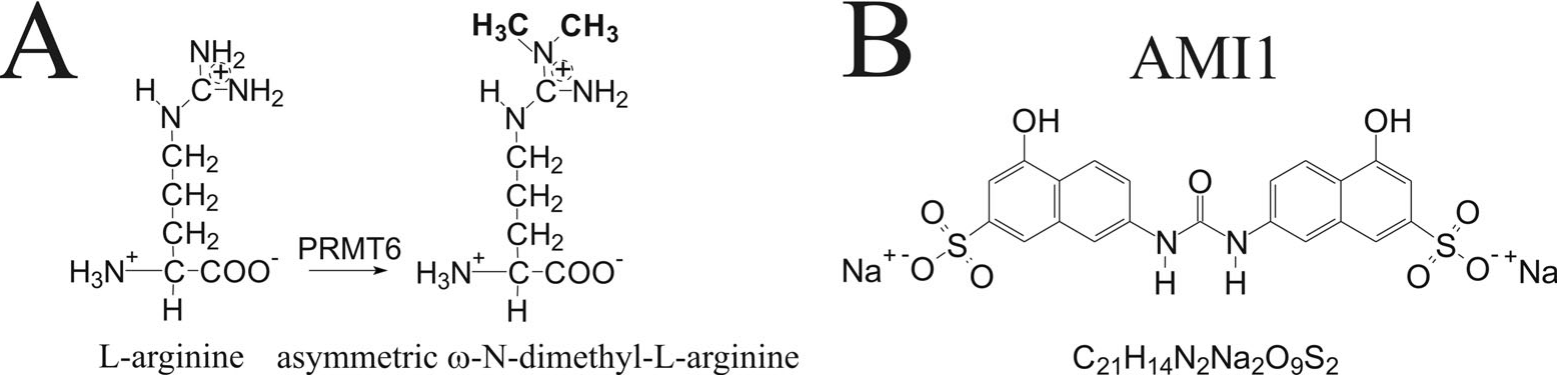
**Asymmetric arginine methylation and structure of AMI1**. *A*, Reaction catalyzed by PRMT6. L-arginine is converted to (asymmetric) ω-N^G^,N^G^-dimethyl-L-arginine by substitution of two hydrogen atoms with two methyl groups in a two step reaction. ω-N^G^-monomethyl-L-arginine is the intermediate. *B*, Structure of AMI1. Standard name: disodium 7,7'-(carbonyldiimino)-bis(4-hydroxy-2-naphthalenesulfonate), M_w_: 548.45.

Arginine methylation is a posttranslational modification that involves the addition of one or two methyl groups to the nitrogen atoms of the guanidino group of arginine [23]. These S-adenosyl-L-methionine-dependent (AdoMet) methylations are carried out by protein arginine methyltransferases (PRMT), a series of enzymes found only in eukaryotes [24]. Arginine methylation has been implicated in RNA processing, transcriptional regulation, signal transduction, and DNA repair, and contributes to the "histone code" [23,25-31]. Two major types of arginine methylation have been described: type I methyltransferases catalyze the formation of ω-N^G^-monomethylarginine and ω-N^G^,N^G^-dimethylarginine (asymmetric); type II enzymes produce ω-N^G^-monomethylarginine and ω-N^G^,N'^G^-dimethylarginine (symmetric) [9,23,25,32]. In humans, nine different PRMTs have been described [23]: PRMT1 [33,34], PRMT3 [35,36], PRMT4 [37], PRMT6 [27] and PRMT8 [38] are all type I enzymes (Fig. 1A), whereas PRMT5 [39,40], PRMT7 [32,41] and PRMT9 [42] are type II enzymes. The classification and activity of PRMT2 [34,43] has not yet been established.

The 41.9 kDa PRMT6 is located in the nucleus and is the only methyltransferase shown to possess automethylation activity [27]. The non-histone chromatin protein HMGA1a is the only host substrate, i.e. not a viral protein, that has been proposed to be methylated by PRMT6 to date [44]. Glycine and arginine rich (GAR) motifs are located in many targets of PRMTs [23,27]; however, all *in vivo *PRMT6 substrates described to date do not seem to be modified at such sites. In regard to the reversibility of arginine methylations, a peptidyl arginine deiminase (PAD4) was shown to have limited arginine demethylating activity, i.e. it is restricted to acting on monomethylarginine [23,45-47].

Some AdoMet analogs were shown to directly inhibit methyltransferases [23]. More recently, a series of small molecules termed arginine methyltransferase inhibitors (AMIs) were shown to act specifically against PRMTs and not to act as competitors of AdoMet. The compound known as AMI1 (Fig. 1B) is cell permeable and inhibits all PRMTs that are active as recombinant proteins [48].

Viral pathogenesis has been related to arginine methylation [23]. For instance, methylation of hepatitis delta virus antigen (S-HDAg) by PRMT1 is essential for RNA replication [49], and methylation of the EBNA1 protein of the Epstein-Barr virus by PRMT1 and PRMT5 is needed for its proper localization to the nucleolus [50]. In addition, hepatitis C virus down-regulates PRMT1 methylation of the helicase of nonstructural protein 3 by increasing expression levels of protein phosphatase 2Ac [51]. Our group demonstrated that HIV-1 Tat is methylated in its ARM by PRMT6 and that this negatively regulates transactivation activity [52]. These findings are also consistent with data on HIV-1 regulation by the transcription elongation factor originally named suppressor of Ty (SPT5), which is methylated by both PRMT1 and PRMT5, showing that an increase in methylation can have a negative impact on viral replication [53]. More recently, it was shown that methylation of viral proteins contributes to maximal levels of viral infectiousness [54].

Yet, it is unknown whether Rev or other viral proteins may also be substrates for PRMT6. Rev harbors an ARM that is very similar to the one found in Tat. However, the ARM of Rev adopts an α-helical structure whereas that of Tat folds as a β-hairpin [16].

Here, we report the arginine methylation of the N-terminal portion of the ARM of Rev by PRMT6. This methyltransferase reduced RRE binding and diminished export of viral RNA to the cytoplasm in cell-based assays. Co-immunoprecipitation experiments confirmed the association of PRMT6 with Rev, which was shown to undergo arginine methylation *in vivo*. Moreover, PRMT6 seemed to attenuate Rev levels, albeit in a manner independent of its methyltransferase activity. These findings demonstrate that PRMT6 impairs HIV-1 Rev protein functions and shed further light on previous observations that PRMT6 can negatively regulate HIV-1 replication.

## Results and discussion

### HIV-1 Rev is specifically methylated by PRMT6

**Figure 2:**
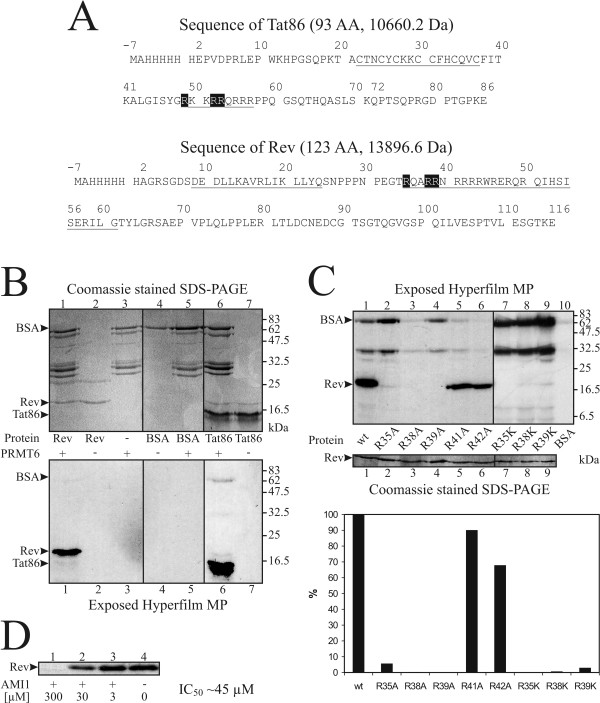
**Specific arginine methylation of Rev by PRMT6 *in vitro***. *A*, Sequences of recombinant histidine-tagged Tat86 and Rev. Both sequences are chimeric and consist of BH10 (amino acids 2–66 and 2–15, respectively) and HXB2 (amino acids 67–86 and 16–116, respectively). Underscored are the cysteine rich motif and the ARM of Tat86, as well as the two α-helices of the helix-loop-helix motif of Rev. Arginine residues located in the N-terminal portion of the ARMs are shaded in black. *B*, Arginine methylation of Rev by PRMT6. Recombinant histidine-tagged Rev was incubated with [*methyl-*^*3*^*H*]-S-adenosyl-L-methionine in the presence (lane 1) or absence (lane 2) of PRMT6. As a positive control, recombinant histidine-tagged Tat86 was incubated with (lane 6) or without (lane 7) PRMT6. As negative controls, BSA was incubated in the presence (lane 5) or absence (lane 4) of PRMT6, or PRMT6 alone was used (lane 3). Proteins were separated by SDS-PAGE, stained with Coomassie blue (upper panel), and tritium incorporation was screened by fluorography (lower panel). The migratory positions are indicated by arrows on the left. *C*, Specific arginine methylation of the N-terminal portion of the ARM of Rev by PRMT6. Recombinant histidine-tagged wild-type (lane 1) and mutant Rev proteins (lanes 2–9), as well as BSA (lane 10) as a negative control, were treated as described in *B*. The Coomassie blue stained gel (center panel) and the developed film (upper panel) were used to calculate the percentages of methylation of the individual mutants (lower panel). The migratory positions are indicated by arrows on the left. Similar results were observed in three experiments. *D*, AMI1 inhibits arginine methylation of Rev by PRMT6. Recombinant histidine-tagged Rev was incubated with PRMT6, as described in *B*, in the presence of increasing amounts of AMI1. Band intensities were quantified to calculate the IC_50_.

The HIV-1 Tat protein contains an ARM that was shown to be a substrate of PRMT6 [52]. Our Rev is chimeric and contains parts of the BH10 (first 15 amino acids) and HXB2 (last 101 amino acids) strains of HIV-1 (Fig. 2A). Sequence comparison between Rev and Tat reveal that the N-terminal portions of their individual ARMs have identical RXXRR motifs. Therefore, it seems logical that the Rev protein may also be a substrate of PRMT6.

To test this possibility, purified histidine-tagged recombinant Rev was incubated together *in vitro *with PRMT6 in the presence of radioactively labeled [*methyl*-^3^H]-S-adenosyl-L-methionine as a methyl donor. As a positive control, we used recombinant histidine-tagged Tat86, and BSA served as a negative control. The proteins were separated by SDS-PAGE, stained with Coomassie blue (Fig. 2B, upper panel), and the labeled proteins were visualized by fluorography (Fig. 2B, lower panel). Rev was shown to be methylated in the presence of PRMT6, whereas no signals were detected in reactions containing only PRMT6 or Rev (Fig. 2B, left). Tat86 gave a positive signal only when PRMT6 was present (Fig. 2B, right). In addition to the intense band of Tat86, there was a weak band visible at the level of PRMT6 due to the previously reported automethylation activity of this methyltransferase [27]. In the case of BSA, no signals were detected (Fig. 2B, center). These findings identify Rev as a substrate of PRMT6, which recognizes sequences different from the GAR motif.

Next, we attempted to map the site of methylation in Rev by mass spectrometry (MS). Measurements by LC/MS resulted in two assigned masses that were 27.8 Da apart in the case of recombinant Rev protein that had been subjected to methylation by PRMT6; this compared well to an expected difference of 28.1 Da in the case of one arginine dimethylation. In contrast, untreated Rev that was not methylated possessed only one mass (Table 1). To map the site, we carried out protease digestions of methylated and untreated Rev to achieve fragmentation. Unfortunately, both glutamyl endopeptidase (Glu-C) and peptidyl-Asp metalloendopeptidase (Asp-N) had limited specificity and many non-specific fragments were generated, yielding inconclusive results when running the LC/MS peptide data through the Mascot (Matrix Science) analyzing software. Furthermore, trypsin could not be used for this analysis, because of justified concerns that it would digest the ARM completely, making mapping impossible. Nevertheless, these data suggest that only one asymmetric arginine dimethylation occurs in Rev.

**Table 1:** Mass of Rev determined by LC/MS

Mass [Da]	Expected	Measured (untreated)	Measured (methylated)
unmodified	13765.4	13763.8	13766.0
1x dimethylation	13793.5	-	13793.8

Left column gives expected values for Rev containing no or one arginine dimethylation. Center column gives measured values for untreated Rev and right column gives measured values for Rev subjected to PRMT6.

Therefore, we chose another strategy to map the methylation site. Namely, we mutated all of the arginine residues of Rev within the N-terminal portion of the ARM. Eight mutants were cloned, each of which contained a single amino acid substitution from R to A or R to K. The eight mutants as well as wild-type Rev were then subjected to PRMT6 methylation, separated by SDS-PAGE, stained with Coomassie blue (Fig. 2C, center panel), and exposed for fluorography as described (Fig. 2C, upper panel). We quantified the bands, taking into account the amount of Rev that had been loaded, with wild-type Rev set at 100% (Fig. 2C, lower panel). The mutant proteins R41A and R42A still produced bands with intensities of 90% and 68% of wild-type Rev, respectively, showing that these residues are not primary substrates of PRMT6. In contrast, the mutant R35A was reduced to a mere 6% of control and R35K was not detectable. The R39A and R39K substitutions resulted in band intensities that were either undetectable or 3% of wild-type, respectively. Methylation of the R38A and R38K mutants was less than 1% in each case.

These findings together with the MS data suggest that one of the three arginine residues at positions 35, 38, and 39 is the methyl acceptor. A possible explanation for the ambiguous result of having more than one target, based on the mutational studies, might be that the two other residues play important roles as part of the recognition motif for PRMT6. Such mutated residues would prevent PRMT6 from binding to Rev and, hence, make arginine methylation of the actual methyl-accepting residue impossible.

Finally, we tested an inhibitor of PRMT6 called AMI1 [48] to see its effects on methylation of Rev (Fig. 2D). Addition of AMI1 abrogated methylation of Rev with an IC_50 _of ~45 μM, showing that AMI1 can inhibit PRMT6 to block arginine methylation of Rev. All these results demonstrate that PRMT6 recognizes Rev as a substrate for specific arginine methylation in the N-terminal portion of the ARM.

### PRMT6 methylates Rev *in vivo *and attenuates Rev protein levels

**Figure 3:**
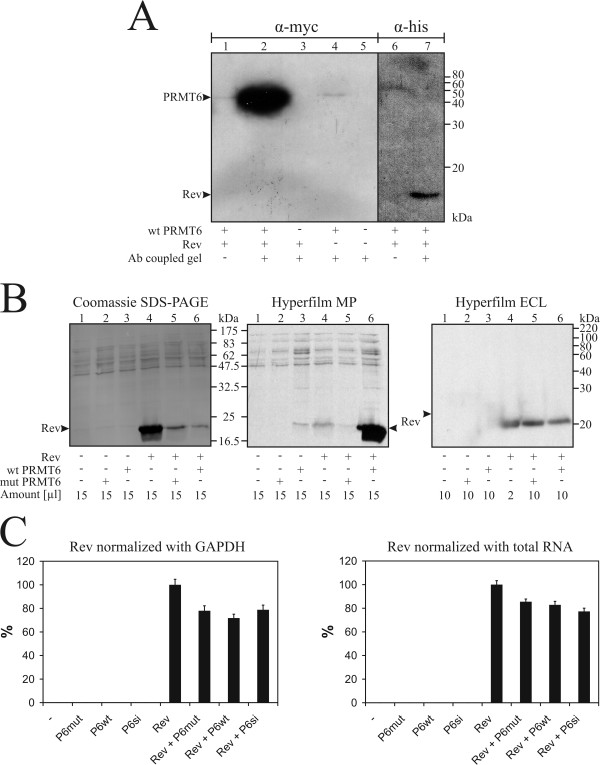
**PRMT6 methylates Rev and attenuates Rev protein levels *in vivo***. *A*, Interaction of Rev and PRMT6. T-REx™-293 cells were transfected with histidine-tagged Rev (lanes 1,2,3,6,7) and myc epitope-tagged PRMT6 (lanes 1,2,4,6,7). Co-IP was carried out with an anti-histidine-tag antibody coupled gel (lanes 2–5,7) and a control gel (lanes 1,6). Eluates were separated by SDS-PAGE, immunoblotted with anti-myc-epitope (lanes 1–5) or anti-histidine-tag antibodies (lanes 6,7) and signals detected with a secondary antibody coupled to HRP. The migratory positions are indicated by arrows on the left. Bottom line: +: antibody coupled gel; -: control gel. *B*, PRMT6 methylates and attenuates Rev *in vivo*. HeLa cells were transfected with histidine-tagged Rev (lanes 4–6) and/or wild-type (lanes 3,6) or mutant (lanes 2,5) myc epitope-tagged PRMT6, or no plasmids (lane 1). After 3 hours pulse labeling, cell lysates were separated by SDS-PAGE, Coomassie stained (left panel) and fluorographed (center panel). Cell lysates were also immunoblotted with anti-Rev antibody and detected as described in *A *(right panel). Loaded amounts of cell lysates are given in μl and the migratory positions are indicated by arrows. *C*, Rev protein levels are not affected by PRMT6 pre-translationally. HeLa cells were transfected as described in *B*. Additionally, HeLa cells expressing siRNA against PRMT6 were used. RNA was isolated for reverse transcription and mean Rev amounts determined by rt-RT-PCR were normalized to GAPDH (left panel) or total RNA (right panel). Rev levels were calculated per amount of Rev in Rev only transfected cells and expressed as percentages. The bars represent standard deviations of the mean of three independent experiments, each of which was carried out in duplicates.

PRMTs are known to interact with their substrates [52]. Therefore, to determine the relevance of our biochemical studies, we wished to assess interaction between PRMT6 and Rev by co-immunoprecipitation (co-IP). T-REx™-293 cells were transfected with plasmids encoding for histidine-tagged Rev and myc epitope-tagged PRMT6. Rev is only expressed upon induction by tetracycline, whereas PRMT6 is under no such control and is continuously expressed. Rev expression was induced at 24 hours after transfection and cells were harvested at 24 hours post-induction. For co-IP we coupled anti-histidine-tag antibody to an activated agarose gel. Cell lysates were co-immunoprecipitated with antibody-coupled gel or a control gel, separated by SDS-PAGE, and immunoblotted with anti-myc-epitope or anti-histidine-tag antibodies (Fig. 3A). The anti-myc-epitope antibody strongly detected PRMT6 in the case of Rev co-transfection (lane 2). Control reactions containing PRMT6, that were purified with a control gel (lane 1) or did not include Rev (lane 4), gave rise to very faint bands, which may represent background of non-specific binding of PRMT6 to the matrix of the gel. No PRMT6 was detected in control reactions in which either PRMT6 (lane 3) or both PRMT6 and Rev (lane 5) were absent. As additional controls, purified cell lysates were visualized with anti-histidine-tag antibody. We detected histidine-tagged Rev with antibody coupled gel (lane 7), but not with a control (lane 6). These findings confirm that Rev and PRMT6 interact and suggest that Rev is a target for PRMT6 *in vivo*.

To prove this, we wished to visualize the extent of Rev methylation by PRMT6 *in vivo*. HeLa cells that had been transfected with Rev and/or PRMT6 (wild-type or a methylation-inactive mutant) were pulse labeled with L- [*methyl-*^*3*^*H*]-methionine. The lysates were separated by SDS-PAGE for subsequent Coomassie staining and fluorography. Lysates were loaded in equal amounts and the Coomassie stain revealed very similar host protein levels when comparisons of the different lanes were enacted. However, there was a very significant difference in Rev protein amounts detected by Coomassie stain (Fig. 3B, left panel). In the case of Rev co-transfected with wild-type PRMT6 (lane 6), the yield of isolated Rev was reduced by 7.5-fold compared to Rev isolated from cells transfected with Rev alone (lane 4). However, Rev co-transfection with mutant PRMT6 (lane 5) also diminished Rev recovery by 5-fold. Hence, comparison of mutant (lane 5) and wild-type PRMT6 (lane 6) revealed a 1.5-fold down-regulation of Rev. Taken together, this suggests the possibility of either decreased expression levels or accelerated degradation of Rev, when co-transfected with PRMT6. However, this Rev attenuation seems mainly due to a still non-defined activity of PRMT6 (5-fold), whereas the methyltransferase activity plays a negligible role (1.5-fold).

In contrast, levels of Rev methylation detected by fluorography were magnitudes higher (Fig. 3B, center panel), i.e. increased by 8-fold, for Rev co-transfected with wild-type PRMT6 (lane 6) compared with Rev transfected alone (lane 4). Taking the attenuation of Rev into account, methyltransferase activity was increased even 60-fold with wild-type PRMT6. As expected, co-transfection with mutant PRMT6 (lane 5) led to 5-fold reduced methylation signals, i.e. no increased methyltransferase activity was detected.

These findings also suggest that the cells used may have low levels of intrinsic PRMT6, since only a fraction of Rev proteins seem to have been arginine methylated under standard conditions. However, in the co-transfection experiment, with increased levels of wild-type PRMT6, virtually all Rev proteins must have been methylated in order to yield such an intense band. The additional bands could not be due to incorporation of labeled methionine during protein synthesis, since relevant amino acids had been omitted from the medium and the drugs cycloheximide and chloramphenicol were employed. Rather, these additional signals originated from methylated proteins modified by the different PRMTs as well as other enzymes that may methylate unrelated proteins. Furthermore, lanes 3 and 6 representing wild-type PRMT6 transfections reveal higher overall signal intensity than the other lanes, although the amounts of protein loaded and visualized by Coomassie staining were the same. Hence, in cells transfected only with wild-type PRMT6 (lane 3), this may explain the weak and sharp band detected at a slightly higher migratory position than the broad band produced by Rev methylation.

Finally, to confirm that the signals indeed originated from Rev, we carried out western blots of the lysates with anti-Rev antibody (Fig. 3B, right panel). The presence of Rev in the lysates from Rev transfected cells (lanes 4–6) was readily visualized, whereas no such signal could be detected in the other lanes. Consistent with the findings of the Coomassie stained gel, the signal produced by Rev-only transfected cells (lane 4) was much more intense than that from co-transfected cells (lanes 5 and 6), when the amounts of protein loaded were compared. Together, these results show that Rev is an *in vivo *target for PRMT6 arginine methylation.

Based on highly different Rev levels in the presence or absence of co-transfected PRMT6, as described above, we designed a real-time reverse transcription polymerase chain reaction (rt-RT-PCR) experiment to assess mRNA levels of Rev under these different transfection conditions (Fig. 3C). This assay clearly distinguishes between pre- or posttranslational regulation of Rev levels by PRMT6 at the level of mRNA or protein. HeLa cells expressing siRNA directed against PRMT6 or mock siRNA were transfected with Rev and/or PRMT6 (wild-type or mutant) as described above and isolated RNA was reverse transcribed. The resulting cDNAs were used to assess mRNA levels of Rev.

Since there is no generally accepted method for normalization of such levels [55-57], we chose two different methods. First, normalization with total RNA amounts obtained from cells was determined by spectrophotometry at 260 nm. Second, normalization was performed using mRNA levels of the house-keeping gene glyceraldehyde-3-phosphate dehydrogenase (GAPDH) by real-time RT-PCR. HeLa cells containing mock siRNA and transfected only with Rev were set at 100% after normalization. The three other samples containing Rev all showed slightly lower mRNA levels independent of the method of normalization employed. In the case of total RNA normalization, the values ranged between 77 and 86%, compared to transfection with Rev alone. Normalization with GAPDH showed slightly lower values in the range of 72 to 79%. As expected, all negative controls did not show any amplification of Rev mRNA.

These results show clearly that the above mentioned 7.5-fold decrease in Rev protein levels is not caused by down-regulation of Rev mRNA by PRMT6. Rather, the decrease in Rev protein is due to the posttranslational interaction of PRMT6 with the Rev protein. However, attenuation is not dependent on methyltransferase activity, but seems to be caused by a yet undefined activity of PRMT6, which may be linked to the proteasome pathway, as previously suggested [58].

### PRMT6 reduces binding of Rev to RRE

**Figure 4:**
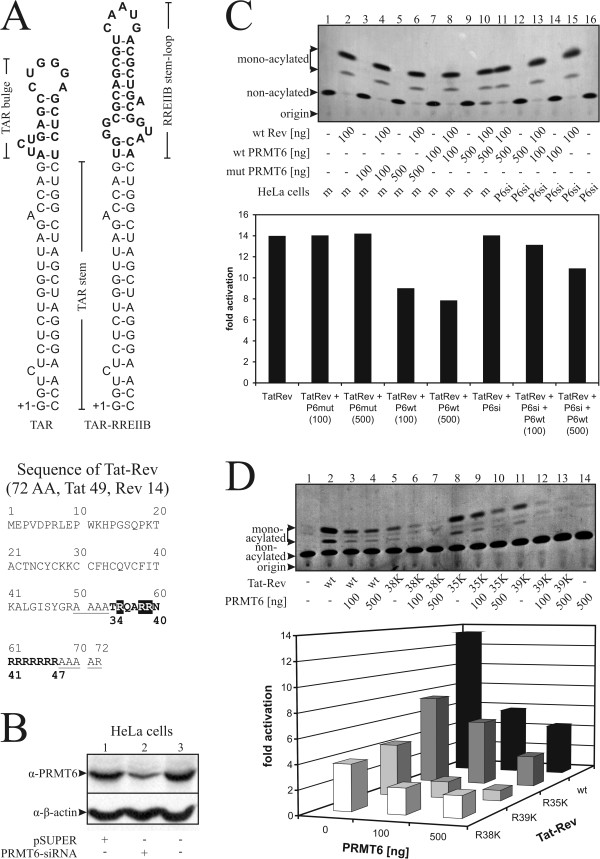
**PRMT6 reduces the interaction between a Tat-Rev fusion protein and a TAR-RREIIB hybrid**. *A*, Sequences of TAR, TAR-RREIIB hybrid and Tat-Rev fusion protein. In TAR-RREIIB, the TAR bulge was replaced by the RREIIB stem-loop (bold). The Tat-Rev fusion protein contains the first 49 amino acids of Tat and is linked to residues 34–47 of Rev (bold) by means of four alanine residues (underscored). Arginine residues changed by mutagenesis are shaded in black. *B*, Knock-down of PRMT6 by pSUPER.retro vector expressing PRMT6-siRNA. HeLa cells expressing PRMT6-siRNA were established using the pSUPER.retro-PRMT6 retroviral vector. Cell lysates were separated by SDS-PAGE and immunoblots were performed. The bands corresponding to PRMT6 protein and the control β-actin are indicated by arrows. *C*, PRMT6 reduces CAT expression due to diminished Rev-RRE interaction. HeLa cells stably transfected with mock siRNA (m, lanes 1–10) or PRMT6-siRNA (P6si, lanes 11–16) were co-transfected with plasmids expressing Tat-Rev (lanes 2,4,6,8,10,11,13,15), pHIV-LTR-RREIIB-CAT (lanes 1–16) and various amounts of myc-tagged PRMT6 (wild-type lanes 7–14, mutant lanes 3–6). At 48 hours post-transfection, CAT assays were performed, separated by TLC and exposed (upper panel). Fold activations, i.e. results of samples (mono-acylated species per total amount of chloramphenicol) divided by those of negative controls without Rev, were calculated from quantified bands (lower panel). The migratory positions are indicated by arrows. Similar results were observed in each of three separate assays. *D*, Mutant R38K is less susceptible to PRMT6 methyltransferase activity. Wild-type (lanes 2–4) and mutated Tat-Rev fusion proteins (R35K lanes 8–10, R38K lanes 5–7 and R39K lanes 11–13) were co-transfected with variable amounts of wild-type PRMT6 into HeLa cells as described in *C *(upper panel). The migratory positions are indicated by arrows. Fold activations were calculated as described in *C *(lower panel). Similar results were obtained in each of three experiments.

Next, we wished to assess whether PRMT6 has any consequences on the interaction of Rev with the RRE *in vivo*. To this end, we used the pHIV-LTR-RREIIB-CAT reporter plasmid, which is derived from the pHIV-LTR-TAR-CAT [59]. RNA transcribed from the latter plasmid is recognized by Tat, which binds to the *trans*-activation responsive element (TAR) and ultimately leads to expression of chloramphenicol acetyltransferase (CAT). In the pHIV-LTR-RREIIB-CAT plasmid, a part of TAR has been replaced by the RREIIB of the Rev response element (Fig. 4A). To obtain optimal binding that leads to high expression of CAT, a Tat-Rev fusion protein is required (Fig. 4A), in which the N-terminal portion of Tat is fused to the ARM of Rev. This ensures maximum binding to the stem-RNA and activates CAT expression.

First, we confirmed knock-down of PRMT6 in HeLa cells that expressed siRNA against PRMT6 (Fig. 4B). Then, levels of expressed CAT were assayed with radioactively labeled [^14^C]-chloramphenicol that becomes mono- or di-acylated in the presence of acetyl-CoA, the linear range showing mono-acylated but no di-acylated species. Reactions separated by TLC were exposed on film and quantified for levels of CAT shown as fold-activation (Fig. 4C). Results with the Tat-Rev fusion protein alone or with Tat-Rev co-transfected with various amounts of mutant PRMT6 all showed activation levels around 14-fold. Furthermore, no apparent effects of siRNA directed against PRMT6 were detected, in part because intrinsic levels of PRMT6 in the HeLa cells used are low. In contrast, co-transfection of Tat-Rev with various amounts of wild-type PRMT6 revealed a PRMT6 dose-dependent reduction of CAT levels by 1.8-fold. As expected, a similar trend was observed in cells expressing siRNA against PRMT6 when co-transfected with wild-type PRMT6, although CAT levels decreased by only 1.3-fold in this circumstance.

Thus, PRMT6 reduces interaction between the ARM of Rev and the RREIIB of the Rev response element, which is most likely due to the methyltransferase activity of PRMT6.

In a second assay, we wished to assess the role of the arginine residues at positions 35, 38, and 39 of the ARM of Rev, one of them being the target for arginine methylation by PRMT6. Therefore, single point mutations were introduced substituting R to K in each case. The results clearly show that all three mutations led to markedly decreased expression of CAT in the absence of PRMT6, indicating that the binding of Tat-Rev to the RRE was considerably reduced (Fig. 4D). Interestingly, the mutant R38K (28%) had the lowest amount of expressed CAT compared to R35K (57%) and R39K (32%), although R38K is not thought to be a main actor in binding to the RRE [16]. This clearly shows that small changes can be very detrimental to good Rev-RRE interaction.

Ideally, the fold-activation of one of these mutants with a substituted lysine instead of the methyl-accepting arginine should be PRMT6-independent; i.e. the absence of a substrate should preclude alterations in RRE binding. When co-expressing different amounts of PRMT6, CAT expression was clearly reduced in a PRMT6-dependent fashion for the wild-type Tat-Rev by 3-fold (Fig. 4D). A similar drop of 3-fold was observed for the R35K mutant, whereas the R39K mutant showed a 5-fold decrease. In the case of R38K, levels of CAT remained at higher levels, corresponding to a 2-fold decrease, meaning that PRMT6 can still reduce RRE binding efficacy, albeit to a lesser extent than for wild-type and the two other mutants.

These results are similar to those of the *in vitro *mutational analysis; i.e. there is no definitive answer as to which of the three residues is the target for arginine methylation by PRMT6. However, the *in vivo *experiments show that interaction between the RRE and the Rev mutant R38K seems to be less dependent on PRMT6 compared to wild-type or other mutant Rev proteins. Therefore, residue R38 is the most likely target of arginine methylation by PRMT6.

### PRMT6 diminishes viral RNA export mediated by Rev

An obvious question is the possible impact of PRMT6 on the export of unspliced or partially spliced viral RNA from the nucleus to the cytoplasm, which is mediated by Rev. To study this, we chose the plasmid pDM128 that contains a portion of HIV-1 proviral DNA in which any HIV-1 genes that are present have been inactivated by mutations [60]. Therefore, the CAT gene, which has been introduced into an intron, is the only gene that is translated into a protein upon Rev-mediated export of the unspliced viral RNA from the nucleus to the cytoplasm.

**Figure 5:**
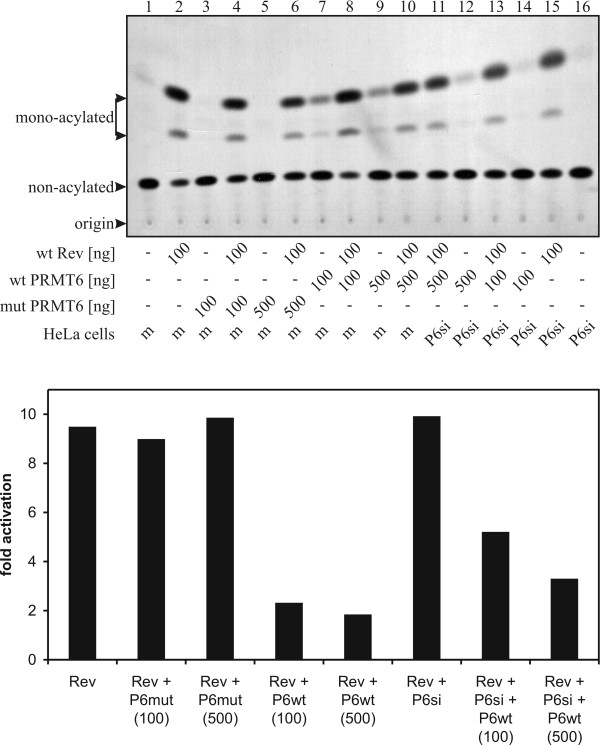
**PRMT6 diminishes Rev mediated viral RNA export**. HeLa cells stably transfected with mock siRNA (m, lanes 1–10) or siRNA against PRMT6 (P6si, lanes 11–16) were co-transfected with pT-REx-DEST30-HRev (lanes 2,4,6,8,10,11,13,15), pDM128 (CAT located in intron, lanes 1–16) and various amounts of myc-tagged PRMT6 (wild-type lanes 7–14, mutant lanes 3–6). At 48 hours post-transfection, CAT assays were exposed (upper panel) and fold activations calculated (lower panel) as described in 4*C*. The migratory positions are indicated by arrows. Similar results were observed in each of three separate assays.

Levels of expressed CAT in transfected HeLa cells were visualized by TLC separation and fold-activations calculated as described above (Fig. 5). Results for Rev alone or Rev co-transfected with various amounts of mutant PRMT6 were all in the same range of 9- to 10-fold activation. siRNA directed against PRMT6 only marginally increased activation upon Rev transfection, which was still around 10-fold, showing that the HeLa cells used apparently express low levels of intrinsic PRMT6, consistent with the results of the experiment described above on Rev-RRE interactions. In contrast, over-expression of wild-type PRMT6 decreased CAT levels by 5-fold in a PRMT6 dose-dependent manner. In the case of wild-type PRMT6, in the presence of siRNA, activation levels were less reduced, i.e. down by 3-fold, compared with results using mock siRNA; the decline was also PRMT6 dose-dependent.

These results demonstrate that diminished RNA export is likely a consequence of the methyltransferase activity of PRMT6.

## Conclusion

We have shown that the HIV-1 Rev protein is a substrate of PRMT6. Mutational and mass spectrometric approaches revealed that a single arginine residue located in the N-terminal portion of the ARM of Rev is the target for PRMT6, with R38 being the most likely methyl-accepting residue. *In vivo *experiments revealed specific association of Rev with the methyltransferase. Furthermore, Rev protein levels were attenuated by both wild-type and a methylase-inactive mutant PRMT6. However, real-time PCR studies did not reveal any specific effects of PRMT6 on mRNA levels of Rev. Thus, Rev protein levels are attenuated posttranslationally by a still non-defined property of PRMT6, independent of its methyltransferase activity. We also demonstrated that only wild-type PRMT6 reduced interaction between Rev and the RRE and, even more important, resulted in diminished Rev-mediated viral RNA export from the nucleus to the cytoplasm. These diminished functions are a direct consequence of the methyltransferase activity of PRMT6. Hence, PRMT6 has a negative impact on HIV-1 Rev function.

Does this mean that arginine methylation of HIV-1 Rev by PRMT6 represents a type of host defense mechanism that can limit rates of viral replication? Reduced binding of Rev to RRE and diminished export rates of unspliced and partially spliced viral RNA are likely detrimental for the virus. However, levels of PRMT6 protein in the cells we have studied are low. Conceivably, methyltransferase activity may be required by the virus to fine-tune different stages of its life cycle. Slightly reduced Rev activity may actually provide benefit to HIV-1 in the context of non-linear Rev function [1] and latency within cells [21,22]. Low levels of Rev protein or low Rev activity, both modulated by PRMT6 methyltransferase activity, led to abrogation of nuclear export of unspliced RNA and may promote proviral latency. This, in turn, could contribute to establishment of latent proviral infection in CD4+ memory T cells. Furthermore, Tat, which was also shown to be a target for PRMT6, has reduced transactivation ability upon PRMT6 methyltransferase activity [52], slowing down transcription of viral RNA, consistent with the latency hypothesis.

A recent report shows that methylation of viral proteins is essential toward attaining optimal levels of HIV-1 infectiousness [54], a conclusion that seems to contradict that revealed in our work. However, the former study employed an inhibitor that blocks all cellular methyltransferase activity, rather than one which specifically interferes only with PRMT6, as we have done. Our findings are more specific than those of Willemsen *et al*. [54], who correctly point out that overall levels of protein methylation are important in viral infectiousness. Thus, these findings, which relate to methylation of both cellular and viral proteins, do not contradict our own in regard to the specific methylation of Rev and other viral proteins by PRMT6.

Inhibitors that target PRMT6 might provide a means of forcing cells to exit latency, similar to inhibition of histone deacetylase (HDAC) by means of valproic acid (VPA) [61]. In this context, AMI1 may be an interesting drug, since it might drive the virus from an early to a late phase of infection, and provide a means of forcing latent proviruses to switch to active replication.

## Methods

### Reagents

Glutathione-S-transferase (GST)-tagged PRMT6 was recloned from pGEX-6P1 [27] into pGEX-4T1 with restriction enzymes *Eco*RI and *Bam*HI. Histidine-tagged Tat86 and myc-epitope-tagged PRMT6 were prepared as previously described [52,62]. Histidine-tagged Rev was cloned from a chimeric HIV-1 cDNA by polymerase chain reaction (PCR) in two stages. In a first step, both exons were amplified separately, whereas the two products were mixed together and amplified with the outer primers in a second reaction. The following primers (Invitrogen) were used: Exon 1 upper primer: 5'-C ACC **ATG **GCG CAT CAC CAT CAC CAT CAC GCA GGA AGA AGC GGA GAC A-3', Exon 1 lower primer: 5'-T GGG AGG TGG GTT GCT TTG ATA GAG AAG CTT GAT GA-3', Exon 2 upper primer: 5'-CTC TAT CAA AGC AAC CCA CCT CCC AA-3', and Exon 2 lower primer: 5'-**TTA CTA **TTC TTT AGT TCC TGA CTC CAA TAC TGT AGG A-3' (start and stop in bold). The PCR products were transferred into the vector pENTR/SD/D-TOPO of the Gateway^® ^System (Invitrogen, Carlsbad, CA, USA) by topoisomerase. These entry vectors were used to transfer the genes into the expression vectors pDEST14 (bacterial expression) and pT-REx-DEST30 (mammalian expression) by LR clonase.

Histidine-tagged mutant Rev proteins were generated with the QuikChange^® ^Site-Directed Mutagenesis Kit (Stratagene, La Jolla, CA, USA) with the following primers (Invitrogen): R35A: 5'-CT CCC AAC CCC GAG GGG ACC GCA CAG GCC CGA AGG AAT AG-3' and 5'-CT ATT CCT TCG GGC CTG TGC GGT CCC CTC GGG GTT GGG AG-3', R38A: 5'-GAG GGG ACC CGA CAG GCC GCA AGG AAT AGA AGA AGA AG-3' and 5'-CT TCT TCT TCT ATT CCT TGC GGC CTG TCG GGT CCC CTC-3', R39A: 5'-GGG ACC CGA CAG GCC CGA GCG AAT AGA AGA AGA AGG-3' and 5'-CCT TCT TCT TCT ATT CGC TCG GGC CTG TCG GGT CCC-3', R41A: 5'-GA CAG GCC CGA AGG AAT GCA AGA AGA AGG TGG AGA G-3' and 5'-C TCT CCA CCT TCT TCT TGC ATT CCT TCG GGC CTG TC-3', R42A: 5'-GA CAG GCC CGA AGG AAT AGA GCA AGA AGG TGG AGA GAG-3' and 5'-CTC TCT CCA CCT TCT TGC TCT ATT CCT TCG GGC CTG TC-3', R35K: 5'-C GAG GGG ACC AAA CAG GCC CGA AGG AAT AG-3' and 5'-CT ATT CCT TCG GGC CTG TTT GGT CCC CTC G-3', R38K: 5'-GGG ACC CGA CAG GCC AAA AGG AAT AGA AG-3' and 5'-CT TCT ATT CCT TTT GGC CTG TCG GGT CCC-3', and R39K: 5'-GA CAG GCC CGA AAG AAT AGA AGA AGA AG-3' and 5'-CT TCT TCT TCT ATT CTT TCG GGC CTG TC-3' (introduced mutations underlined).

The Tat-Rev fusion protein was described earlier [59]. Mutant Tat-Rev fusion proteins were also generated with the QuikChange^® ^II XL Site-Directed Mutagenesis Kit with the following primers (Invitrogen): R35K: 5'-GT GCC GCT GCA GCC ACC AAA CAG GCC AGG CGA AAC AG-3' and 5'-CT GTT TCG CCT GGC CTG TTT GGT GGC TGC AGC GGC AC-3', R38K: 5'-C GCT GCA GCC ACC AGA CAG GCC AAG CGA AAC AGG AGA C-3' and 5'-G TCT CCT GTT TCG CTT GGC CTG TCT GGT GGC TGC AGC G-3', and R39K: 5'-C ACC AGA CAG GCC AGG AAA AAC AGG AGA CGG CGA CGT C-3' and 5'-G ACG TCG CCG TCT CCT GTT TTT CCT GGC CTG TCT GGT G-3' (introduced mutations underlined).

The pHIV-LTR-RREIIB-CAT reporter plasmid is derived from the pHIV-LTR-TAR-CAT [59]. Briefly, a portion of TAR has been replaced by the RREIIB, which binds strongly to Tat-Rev fusion proteins and ultimately leads to expression of CAT. The pDM128 reporter plasmid, a kind gift from the laboratory of Dr. Alan Frankel, UCSF, San Francisco, CA, USA, was described earlier [60]. Briefly, the CAT gene is located in an intron and is only expressed upon export of the unspliced RNA by Rev, which binds to RRE.

Anti-histidine-tag (Rb), anti-Rb IgG (Gt, HRP coupled), anti-myc-epitope (Mo) and anti-Mo IgG (Sh, HRP coupled) antibodies were purchased from United States Biological (Swampscott, MA, USA). Anti-Rev (Rb) antibody was a kind gift from the laboratory of Dr. Alan Cochrane, University of Toronto, Toronto, Canada.

AMI1, a kind gift from the laboratory of Dr. Mark T. Bedford, University of Texas, Smithville, TX, USA, was solubilized in milliQ H_2_O at a concentration of 10 mM.

### Methylation assays

1–2 μg of recombinant histidine-tagged Rev (wild-type or mutated), Tat86, or bovine serum albumin (BSA) (New England Biolabs, Pickering, ON, Canada) were incubated with 3–4 μg GST-tagged PRMT6 in the presence or absence of AMI1 together with 0.55 μCi of [*methyl-*^*3*^*H*]-S-adenosyl-L-methionine (Perkin Elmer life sciences, Boston, MA, USA) and TE buffer (1.67 mM Tris, 0.33 mM EDTA, pH 7.4) for 3 hours at 37°C in a final volume of 10 μl. Reactions were stopped by adding 10 μl of 2 × Lämmli buffer (Bio-Rad Laboratories, Hercules, CA, USA), followed by boiling for 5 minutes and centrifugation at 16,000 *g *for 2 minutes. Samples were loaded on 15% polyacrylamide gels containing sodium dodecyl sulfate (SDS) and a high level of *N,N,N',N'*-tetramethylethylenediamine (TEMED). Gels were stained with Coomassie brilliant blue R-250 solution (Bio-Rad Laboratories) and, after destaining, soaked in Amplify (Amersham Biosciences, Little Chalfont, Buckinghamshire, UK) for 30 minutes. Gels were dried and exposed for fluorography on Hyperfilm MP (Amersham Biosciences) for 1 to 3 days. Gels and films were quantified with GeneTools (SynGene) and the IC_50 _for AMI1 were calculated with Prism 4 (GraphPad Software Inc.).

### Mass spectrometry

Methylated or untreated recombinant histidine-tagged Rev was analyzed by liquid chromatography/mass spectrometry (LC/MS). Intact protein reaction mixtures were first separated by LC on a C18 reversed phase biobasic picofrit column (New Objective) and then injected into a Q-Trap 4000 (Sciex-Applied Biosystems, Concord, ON, Canada). Molecular weight determinations were carried out with BioAnalyst software that is part of Analyst 1.4.

### Co-immunoprecipitation

Experiments were carried out with the ProFound™ Mammalian Co-Immunoprecipitation Kit (Pierce, Rockford, IL, USA) in accordance with manufacturer's instructions. Briefly, anti-histidine-tag antibody was coupled to an amine reactive agarose gel, whereas a control gel was generated by using buffer instead of antibody. 60 μl Lipofectamine 2000 per 1.5 ml of Dulbecco's modified Eagle medium (DMEM) were mixed with 1.5 ml DMEM containing 0 or 12 μg pT-REx-DEST30-HRev, and 0 or 12 μg pVAX-mycPRMT6 (wild-type or methylase-inactive mutant), and variable amounts (adjust total DNA to 24 μg) of pGEM for transfection into T-REx™-293 cells (Invitrogen). At 24 hours post-transfection, Rev expression was induced by adding tetracycline to a final concentration of 1 μg/ml. After 24 hours, cells were harvested and lysed. Cell lysates were bound to coupled or control gels, washed, and bound proteins were eluted. Eluates were separated on 15% polyacrylamide gels containing SDS and high TEMED. Gels were immunoblotted with anti-myc-epitope or anti-histidine-tag antibodies and detected with secondary antibodies coupled to HRP using the ECL™ Plus western blotting detection system on Hyperfilm ECL™ (Amersham Biosciences).

### *In vivo *methylation

Similar experiments were described earlier [52]. Briefly, HeLa cells were transfected with pT-REx-DEST30-HRev and/or pVAX-mycPRMT6 (wild-type or mutant). At 24 hours post-transfection, the cells were pulse labeled with L- [*methyl-*^*3*^*H*]-methionine (Amersham Biosciences) for 3 hours in the presence of cycloheximide (100 μg/ml) and chloramphenicol (40 μg/ml) (both Sigma) in DMEM lacking the amino acids methionine, cysteine and glutamine to prevent protein synthesis. The cells were lysed in RIPA buffer (150 mM NaCl, 50 mM Tris, 1% NP40, 0.5% deoxycholic acid, 0.1% SDS, pH 8) containing complete mini EDTA-free protease inhibitor (Roche) and processed as described for the methylation assays. Rev was confirmed by western blot with anti-Rev antibody detected with an anti-Rb antibody conjugated to HRP as described above.

### Real-time RT-PCR

10^6 ^HeLa cells stably transfected with siRNA against PRMT6 [52] or empty pSUPER vector (Oligoengine, Seattle, WA, USA) were seeded in 6 cm plates and incubated for 24 hours. 20 μl Lipofectamine 2000 per 0.5 ml DMEM were mixed with 0.5 ml DMEM containing 0 or 3 μg pT-REx-DEST30-HRev, 0 or 3 μg pVAX-mycPRMT6 (wild-type or mutant), and variable amounts (adjust total DNA to 9 μg) of pGEM for transfection. At 24 hours post-transfection, cells were harvested and RNA isolated with the RNeasy^® ^Protect Mini Kit (Qiagen) according to manufacturers instructions, including on-column DNase I digestion. 500 ng isolated RNA were used to synthesize cDNA with the QuantiTect^® ^Reverse Transcription Kit (Qiagen) according to the manual.

Real-time PCR was carried out on a Rotor-Gene RG 6000 (Corbett Research, Sydney, Australia) using the RealMasterMix (2.5x) Kit (Eppendorf). In three independent experiments, samples were measured in duplicates and standards in triplicates. Briefly, cDNAs (25 ng of initial RNA in RT reactions) were added to the master mix containing 0.5 μM of each Rev primer (5'-GAC CTC CTC AAG GCA GTC AGA-3' and 5'-CGC AGA TCG TCC CAG ATA AGT-3', purchased from IDT, Coralville, IA, USA) in a final volume of 20 μl. As standard, 2 × 10^2 ^to 2 × 10^7 ^copies/reaction of linearized pT-REx-DEST30-HRev were used. After an initial denaturing step of 95°C for 2 min, 40 cycles of 95°C for 5 s, 48°C for 15 s, and 68°C for 15 s were performed, followed by a melting curve. To normalize Rev levels we chose two different approaches: 1) isolated total RNA amounts were determined on a BioPhotometer 6131 (Eppendorf) at 260 nm, or 2) GAPDH levels were determined with the Hs_GAPDH_+_SG QuantiTect^® ^Primer Assay (Qiagen) and the qPCR Plasmid Standard (High Abundance) (Invitrogen) with 2 × 10^2 ^to 2 × 10^7 ^copies/reaction. After an initial denaturing step of 95°C for 3 min, 40 cycles of 95°C for 10 s, 55°C for 15 s, and 68°C for 15 s were performed, followed by a melting curve. Mean Rev levels were normalized according to mean GAPDH levels or total RNA amounts and quantities were expressed in relation to the mean value measured in mock siRNA HeLa cells transfected only with Rev.

### Generation of HeLa cells stably expressing siRNA against PRMT6

Oligonucleotides encoding siRNAs directed against PRMT6 mRNA have been described previously [52] and were purchased from Invitrogen (CA, USA). These oligonucleotides were annealed and ligated into pSUPER.retro (Oligoengine) downstream of the H1 promoter, giving rise to the pSUPER.retro-PRMT6 retroviral vector. The latter was used to transfect Phoenix packaging cells with Lipofectamine 2000 (Invitrogen) to produce ecotropic retroviral supernatants, which, at 48 hours post-transfection, were filtered through a 0.45 μm filter. This filtrate was then used to infect HeLa cells, and infected cells were selected with puromycin (2 μg/ml) for two weeks. Stable knock-down of the PRMT6 gene was determined by western blot analysis to ensure that knock-down, mediated by pSUPER.retro, was maintained over long periods.

### CAT assay for Rev-RRE interaction

5 × 10^5 ^HeLa cells (PRMT6- or mock siRNA) were seeded in 12-well plates and incubated for 24 hours. 4 μl Lipofectamine 2000 (Invitrogen) per 0.1 ml DMEM were mixed with 0.1 ml DMEM containing 100 ng pHIV-LTR-RREIIB-CAT, 100 ng pSV2/TatRev (wild-type or mutated), variable amounts (100/500 ng) of pVAX-mycPRMT6 (wild-type or mutant), and variable amounts (adjusted to 2 μg total DNA) of pGEM for transfection. At 48 hours post-transfection, cells were harvested in 800 μl TEN buffer (40 mM Tris, 1 mM EDTA, 150 mM NaCl, pH 7.5), centrifuged, and cell pellets were resuspended in 80 μl Tris (250 mM, pH 7.5) to carry out 3 freeze-thaw cycles. 50 μl of cell lysate were mixed with 2 μl [^14^C]-chloramphenicol (100 μCi/ml, Amersham), 20 μl acetyl-CoA (10 mg/ml, Sigma) all in Tris (250 mM, pH 7.5) in a final volume of 150 μl and incubated for 1 hour at 37°C. 450 μl ethyl acetate were added, mixed, centrifuged and 400 μl of the upper ethyl acetate phase were dried for 15 minutes in a Speed-vac centrifuge. Pellets were solubilized in 18 μl ethyl acetate, separated by thin layer chromatography (TLC) (20 × 20 cm, Merck) and exposed to Hyperfilm MP.

### CAT assay for Rev-mediated RNA export

5 × 10^5 ^HeLa cells (PRMT6- or mock siRNA) were seeded and transfected as described above, replacing pHIV-LTR-RREIIB-CAT and pSV2/TatRev by pDM128 and pT-REx-DEST30-HRev, respectively. CAT isolation, reaction and separation was carried out as described above.

## Authors' contributions

CFI carried out all work presented in the figures with one exception noted below. CFI also drafted the manuscript. BX cloned and isolated Tat86, PRMT6, and wild-type Tat-Rev fusion protein, and stably transfected HeLa cells with siRNA directed against PRMT6 or mock siRNA, and carried out the experiments for figure 4B. SR provided the initial clones of PRMT6, helped in concepts and design of *in vitro *assays, and was involved in revising the manuscript. MAW participated in the concept and design of the experiments, and critically revised the manuscript. All authors have read and approved the final manuscript.
